# Comparative effectiveness of direct admission and admission through emergency departments for children: a randomized stepped wedge study protocol

**DOI:** 10.1186/s13063-020-04889-9

**Published:** 2020-11-30

**Authors:** JoAnna K. Leyenaar, Corrie E. McDaniel, Stephanie C. Acquilano, Andrew P. Schaefer, Martha L. Bruce, A. James O’Malley

**Affiliations:** 1 Department of Pediatrics, Children’s Hospital at Dartmouth-Hitchcock Medical Center, Lebanon, New Hampshire USA; 2 The Dartmouth Institute for Health Policy & Clinical Practice, Geisel School of Medicine at Dartmouth College, Hanover, NH USA; 3 Department of Pediatrics, University of Washington, Seattle Children’s Hospital, Seattle, WA USA; 4 Department of Psychiatry, Dartmouth-Hitchcock Medical Center, Lebanon, NH USA; 5 Department of Biomedical Data Science, Geisel School of Medicine at Dartmouth College, Hanover, NH USA

**Keywords:** Implementation, Direct admission, Cluster randomized controlled trial, Pediatric hospitalizations, Multi-stakeholder teams, Pediatric hospital medicine

## Abstract

**Background:**

Approximately 2 million children are hospitalized each year in the United States, with more than three-quarters of non-elective hospitalizations admitted through emergency departments (EDs). Direct admission, defined as admission to hospital without first receiving care in the hospital’s ED, may offer benefits for patients and healthcare systems in quality, timeliness, and experience of care. While ED utilization patterns are well studied, there is a paucity of research comparing the effectiveness of direct and ED admissions. The overall aim of this project is to compare the effectiveness of a standardized direct admission approach to admission beginning in the ED for hospitalized children.

**Methods/design:**

We will conduct a stepped wedge cluster randomized controlled trial at 3 structurally and geographically diverse hospitals. A total of 70 primary and urgent care practice sites in the hospitals’ catchment areas will be randomized to a time point when they will begin participation in the multi-stakeholder informed direct admission program. This crossover will be unidirectional and occur at 4 time points, 6 months apart, over a 24-month implementation period. Our primary outcome will be the timeliness of clinical care provision. Secondary outcomes include (i) parent-reported experience of care, (ii) unanticipated transfer to the intensive care unit within 6 h of hospital admission, and (iii) rapid response calls within 6 h of hospital admission. We anticipate that 190 children and adolescents will be directly admitted, with 1506 admitted through EDs.

Analyses will compare the effectiveness of direct admission to admission through the ED and will evaluate the causal effect of implementing a direct admission program using linear regression with random effects for referring practice clusters and time period fixed effects. We will further examine the heterogeneity of treatment effects based on hypotheses specified a priori. In addition, we will conduct a mixed-methods process evaluation to assess reach, effectiveness, adoption, implementation, and maintenance of our direct admission intervention.

**Discussion:**

Our study represents the first randomized controlled trial to compare the effectiveness of direct admission to admission through the ED for pediatric patients. Our scientific approach, pairing a stepped wedge design with a multi-level assessment of barriers to and facilitators of implementation, will generate valuable data about how positive findings can be reproduced across other healthcare systems.

**Trial registration:**

ClinicalTrials.gov NCT04192799. Registered on December 10, 2019).

## Contributions to the literature

- This study represents the first randomized controlled trial to compare the effectiveness of direct admission to emergency department admission.
- By examining multi-stakeholder informed admission processes and outcomes across diverse settings, this study will expand our understanding of the factors that support and hinder hospital admission processes.
- The multi-level assessment of barriers to and facilitators of study implementation will generate valuable data about how positive findings can be reproduced in other healthcare systems.

## Background

Approximately two million children are admitted to hospitals in the United States (US) each year [1]. These hospitalizations are costly, incurring healthcare costs that represent 40% of all national pediatric healthcare expenditures [2, 3]. In addition, hospital admissions have major implications for patients’ and families’ quality of life, disrupting normal routines, resulting in time lost from work and school, and contributing to both financial and emotional stress [4]. These stressors are disproportionately experienced by children with chronic illnesses and their families, who account for more than 50% of pediatric hospital admissions in the US [1].

Over the last decade, national programs have dedicated significant resources to improving hospital discharge processes: the Agency for Healthcare Research and Quality supported a major initiative to re-engineer discharge practices, the Centers for Medicaid and Medicare transformed payment structures with a focus on hospital readmissions, and national physicians organizations developed a Transitions of Care Consensus Policy Statement [5–7]. Accordingly, the phrase “transition of care” is widely understood to describe the changes in setting, healthcare providers, and disease management strategies experienced by hospitalized patients at the time of discharge. Like hospital discharge, hospital admission involves transitions in sites of care, handoffs between healthcare providers, and changes in medical therapies. Both are associated with significant stress to patients and their families [8, 9]. While hospital discharge processes have been the focus of tremendous research, policy, and quality improvement efforts, research determining the quality of care at the time of hospital admission is scant [5, 9, 10]. As a result, hospital admissions expose patients to many of the same risks that have been the focus of hospital discharge reform: unstructured patient handoffs, poor communication between healthcare providers, and inefficient care [5–7].

In the US, patients in need of hospital admission enter the hospital through one of two primary mechanisms: via the emergency department (ED) or via direct admission, defined as admission to hospital from the community without first receiving care in the hospital’s ED. Over the last two decades, EDs have increasingly served as portals for hospital admission, contributing to care fragmentation and ED crowding [11]. In a landmark report, the Institute of Medicine describes our emergency medical system as overburdened, fragmented, and at the breaking point [12]. Yet, of the 1.5 million non-elective pediatric hospitalizations that occur each year, 75% originate in EDs with the remainder occurring via direct admission [13].

While ED utilization patterns have been well studied, there is a paucity of research comparing the effectiveness of direct and ED admissions, particularly in children [11, 14, 15]. Direct admission may offer benefits for both patients and healthcare systems, including reduced ED volumes, improved coordination between outpatient and hospital-based healthcare providers, and improved family experience of care. Currently, direct admission rates vary substantially across hospitals and conditions, with condition-specific direct admission rates for unplanned hospitalizations ranging from 9% for appendectomy to 38% for bipolar disorder [13]. These quantitative data are mirrored in a national survey of pediatric medical directors reporting direct admission rates at their hospitals that range from less than 10% to greater than 50% [16]. Despite this, only one-third report having formal direct admission policies in place, while 50% report a belief that more children should be admitted directly. This significant variation across hospitals and conditions indicates clinical uncertainty regarding hospital admission best practices. We therefore propose a multi-site stepped wedge randomized controlled trial to (i) compare the effectiveness of direct admission to admission through the ED and (ii) evaluate the causal effect of implementing a direct admission program.

## Methods

### Aims

The overall goal of this project is to compare the effectiveness of a standardized direct admission approach to admission beginning in the ED for hospitalized children. We will achieve this goal by addressing the following specific aims: (1) determine the effect of a pediatric direct admission intervention on timeliness of healthcare provision, family experience of care, and rates of clinical deterioration compared to pediatric hospital admission beginning in the ED; (2) identify the pediatric populations and conditions that experience the greatest benefits from direct admission with respect to timeliness of healthcare provision and family experiences of care; and (3) identify barriers to and facilitators of implementing direct admission processes by applying qualitative and quantitative methods.

For aim 1, we hypothesize that a direct admission will be associated with more rapid initiation of clinical care and improved family experience of care with no significant differences in rates of clinical deterioration compared with admission beginning in the ED. Further, we hypothesize that by making direct admission to hospital from a primary or urgent care practice available, we will reduce the average time until initiation of clinical care across all patients (whether admitted directed or via the ED). For aim 2, we hypothesize that children with urinary tract infections (UTIs) or skin and soft tissue infections (SSTIs) will experience the greatest benefits from direct admission relative to other conditions examined, as these conditions generally require minimal diagnostic testing or specialized equipment prior to treatment initiation. We further hypothesize that children with complex chronic conditions will experience greater benefits than children without these conditions, as they are often well known to the hospital-based healthcare teams and may be more likely to receive timely, personalized care. Underlying these hypotheses is our theory that, by eliminating ED wait times and exposure to the often hectic ED environment, and by reducing the number of healthcare teams involved in care provision, directly admitted children will have reduced time to clinical care and improved experiences.

### Theoretical framework

**Fig. 1:**
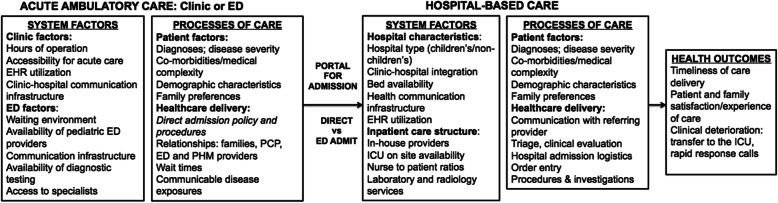
Conceptual framework informing research approach. *Donabedian A. Evaluating the quality of medical care. *Milbank Mem Fund Q*. 1966;44(3):166S–206S

Direct admission to hospital has several prerequisites, including access to timely ambulatory care for children with acute illnesses and communication between outpatient and inpatient healthcare providers to initiate direct admission referrals. Our conceptual framework is modified from Donabedian’s structure, process, outcome framework to also incorporate patient characteristics and family preferences (Fig. 1) [17]. This framework illustrates how systems factors and processes of care may influence where children access ambulatory care for acute illnesses (ED, urgent care centers, or primary care clinics) and whether admission occurs directly or through the ED. These factors, in turn, influence health outcomes. The system factors summarized in this model, including clinic and ED factors, were described by parents of hospitalized children as influential to their decisions about when and where they seek care for their child’s acute illnesses [8]. The processes of care factors are derived from deliberative discussions with stakeholders in the hospital admission process [18].

### Design

**Fig. 2:**
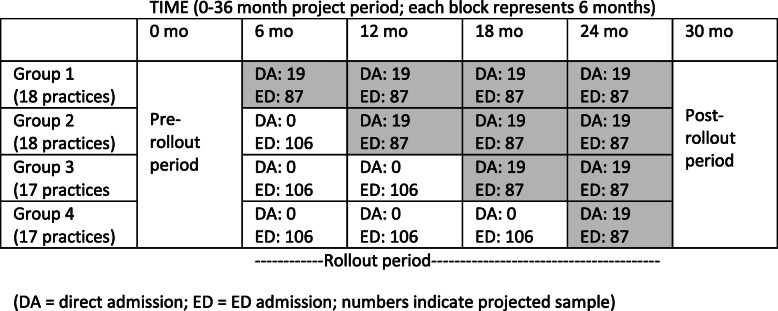
Characteristics of stepped wedge cluster randomized controlled trial, where shaded areas indicate direct admission intervention exposure and unshaded areas indicated control conditions. DA, direct admission; ED, ED admission. Numbers indicate projected sample

We will conduct a cluster randomized controlled trial (RCT) at 3 structurally and geographically diverse hospitals to compare the effectiveness of direct admission to admission through the ED and to evaluate the causal effect of implementing a direct admission program. To perform these analyses, we will randomize 70 primary and urgent care practice sites in the hospitals’ catchment area to a time point for crossing over to the direct admission intervention (Fig. 2); leadership at each site will agree to randomization prior to project start. This crossover will be unidirectional and occur at 4 time points, 6 months apart, over the 24-month implementation period. Practices in each group will be randomized using a stratified approach, with 17–18 practice sites (4–9 practice sites per hospital) randomized to crossover to the direct admission intervention at each step/crossover point, stratified by hospital and practice group, with the computer-generated random allocation conducted by AJO. In preparing this protocol report, we followed Consolidated Standards of Reporting Trials extension for Cluster Trials and the Standard Protocol Items: Recommendations for Interventional Trials (SPIRIT) Guidelines. (Additional Files 1 and 2) [19].

### Setting

The study will be carried out at three health systems in the US, selected for their structural diversity, current low direct admission rates, and strong institutional support to implement and evaluate direct admission processes. The first system has a freestanding children’s hospital with a pediatric ED annual volume of over 80,000 visits and a pediatric hospital medicine (PHM) service that provides care to more than 9000 children each year. At this site, four ambulatory care groups will participate in the intervention including one practice group owned by the hospital system and three practice groups affiliated with the hospital through a quality of care alliance. Across these four ambulatory care groups, 34 unique primary and urgent care practice sites will be randomized to participate in this study. The second participating health system also has a freestanding children’s hospital with a pediatric ED annual volume greater than 90,000 visits, a PHM service that provides care to 6000 children each year, and a pediatric infectious disease service that admits approximately 3400 hospitalizations yearly. At this hospital, there are 18 ambulatory care partners, including 12 primary care practices and 6 urgent care centers, all of which are owned by the health system. Our third system has a general hospital where children seeking care in their ED are cared for in a general (non-children’s) ED by general (non-pediatric fellowship-trained) emergency medicine physicians. This hospital admits approximately 650 children annually and the ambulatory care partners at this site include three primary care groups who practice across 18 unique primary care sites and affiliated urgent care centers.

### Comparators and study eligibility

At the patient level, we will evaluate the comparative effectiveness of direct versus ED admission. At the system level, we will compare outcomes in children who are admitted from eligible primary and urgent care practices where the direct admission intervention is available (including children admitted directly and through EDs) to those admitted from practices not yet eligible for the direct admissions intervention (only ED admission is available). Eligible children will be nested within their respective primary care practices. We will limit our analysis to children < 18 years of age admitted with the following common medical reasons for hospitalization: gastroenteritis, dehydration, SSTI, UTI/pyelonephritis, pneumonia, influenza, and viral infection not otherwise specified. These diagnoses comprise approximately 25% of all unscheduled pediatric admissions nationally and represent conditions that have been identified as appropriate for direct admission but not currently accepted for direct admission at the participating hospitals [1, 18]. Children with planned admissions (e.g., chemotherapy), those admitted to the intensive care unit or other subspecialty services, and those transferred from other hospitals will be excluded.

### Intervention core elements

The direct admission intervention will involve five core elements, endorsed by a national multi-stakeholder panel: [18] (i) direct admission education and tools for both referring healthcare providers (e.g., primary care physicians (PCPs)) and accepting healthcare providers (nurses, resident physicians and pediatric hospitalists), including diagnoses and populations eligible for direct admission, and patient referral methods; (ii) a system to facilitate direct communication between referring and accepting healthcare providers, enabling a single telephone call from a referring physician to an accepting hospital-based physician (currently in place at all hospitals to facilitate acceptance of inter-hospital transfers), and use of a structured data collection tool to facilitate determination of the appropriateness of the patient for direct admission; (iii) instructions for families regarding when/where/how to proceed for direct admission; (iv) rapid evaluation of clinical stability of the patient upon hospital arrival by the inpatient healthcare team; and (v) timely initiation of appropriate clinical care.

### Local adaptation and leadership teams

Each site will develop a multi-stakeholder Direct Admissions Leadership Team (DALT), comprised of key stakeholders in the hospital admission process including pediatric hospitalists, nurses, patient and family representatives, PCP representatives, and hospital administrators. During the pre-implementation period, each DALT will meet to discuss intervention elements within the context of local system, organization, provider, and patient characteristics. These discussions will be supported by review of local administrative data and pilot data of study outcomes, with tailoring of educational materials and tools as appropriate. During the implementation phase, the DALT and central research team will meet to discuss implementation fidelity and the need for potential adaptation.

### Outcome measures and data collection

Our primary outcome is timeliness of clinical care provision, defined as the time from arrival at the hospital until initiation of clinical care by the accepting healthcare provider (including diagnostic testing and/or medical management). This outcome will be derived from electronic health record (EHR) time stamps; all project sites use EHRs to document times of patient registration, orders, administration of medications and therapies, and diagnostic tests. EHR time stamps have been previously validated using time motion data [20]; during the pre-implementation period, we will similarly validate the accuracy of time stamps at each hospital.

Secondary outcomes include (i) parent-reported family experience of care; (ii) unanticipated transfer to the intensive care unit (ICU) within 6 h of hospital admission, a marker of clinical deterioration [21]; and (iii) rapid response calls within 6 h of hospital admission, a second marker of clinical deterioration defined as calls to the hospital’s medical-emergency team placed by any person concerned about signs of critical clinical deterioration.

Family experience of care will be determined by parent-report using an instrument modified from existing experience of care measures, including the Child Hospital Consumer Assessment of Healthcare Providers and Systems Survey (Child HCAHPS) [22] and the Consumer Quality Index [23]. Following instrument development and pilot testing, this parent-reported outcome will be administered via electronic tablet to parents/guardians between 6 and 72 h following hospital admission.

### Predictor variables

We will evaluate, at baseline, patient-level characteristics that may be associated with the portal of hospital admission. These include child age, gender, race/ethnicity, primary payer (Medicaid, commercial, other), primary reason for hospital admission (clinical condition), medical complexity, and level of care (observation or inpatient status). Medical complexity will be categorized as no complex chronic conditions, one complex chronic condition (i.e., respiratory disease), or two or more complex chronic conditions (i.e., respiratory and neuromuscular disease) by applying a previously established algorithm [24].

### Analytic plan

For aim 1, we will conduct two analyses. The first analysis will compare the effectiveness of direct and ED admission at the patient level using an observational approach. The second analysis will evaluate our hypothesis that the availability of a direct admission program will be associated with an overall increase in the rapidity of initiation of clinical care and improved family experience of care with no significant differences in rates of clinical deterioration compared to not having direct admission available. In this second analysis, due to randomization, we expect the distributions of patient characteristics to be balanced between practices eligible for direct admission and those not yet randomized to the direct admission program. However, the cluster-randomized structure of the study allows for the possibility that non-trivial imbalances in patient covariates could occur between the study arms. Therefore, prior to analyzing any outcomes, we will compare the distribution of baseline covariates between the groups. We will then examine unadjusted differences in our outcomes. To adjust for observed predictors related to direct and ED admission, including temporal trends, for our main analyses, we will use regression models. Because all patients receive some form of clinical care at the hospital irrespective of the form of admission, there is no censoring associated with the measurement of time to clinical care. As a result, standard regression models may be used.

#### Analysis I: comparative effectiveness of direct and ED admission

The statistical model for our patient-level comparison of direct admission versus ED admission is an observational analysis due to patients not being randomly assigned to admission type. This model (i) is represented as:

$$ {Y}_{ijht}={\beta}_0+{\beta}_1D{A}_{ijht}+{\beta}_2{x}_{ijht}+{\beta}_{3t}+{\beta}_{4h}+{\theta}_{jh}+{\varepsilon}_{ijht} $$

where *Y*_*ijht*_ is the outcome variable and *DA*_*ijht*_ is a binary variable indicating whether the patient was admitted via direct admission (*DA*_*ijht*_ = 1) or via the ER (*DA*_*ijht*_ = 0) for patient *i* seen by clinic *j* at hospital *h* at time *t*. In addition, *x*_*ijht*_ is a vector of patient-level controls and *θ*_*jh*_ is a random effect for clinic within hospital. The model also includes fixed-effects for time-period, *β*_3*t*_, and hospital, *β*_4*h*_, to capture the general unstructured trend across calendar time and hospital-specific effects, respectively. The key coefficient of interest is *β*_1_, which captures the association of a patient being admitted directly versus via the ED. In order to avoid contamination from outcomes of ED observations when direct admission was not available, for this analysis, we plan to only use observations from the post-periods. However, to gain some insight into the closeness of the results from this analysis with those that we would expect to see if it was possible to have patient-level randomization, in a supplemental analysis, we will include the pre-intervention ED observations and test if there is a change in the mean ED outcome in the post-intervention period. A finding of minimal change over time will support a hypothesis that, in the absence of the intervention, the outcomes for direct admission patients would have been the same as those observed for patients admitted through the ED.

### II. Causal analysis

In addition to the above-described patient-level analysis, we will analyze the data under the cluster randomized stepped wedge design to estimate the causal effect of the availability of direct admission on our primary outcome, time to receipt of clinical care. The key predictor in this analysis is a clinic-level time-varying predictor indicating whether or not direct admission is available, with its coefficient capturing the effect of direct admission availability on the average time until clinical care is received across all patients, whether or not they were directly admitted. The statistical model for this analysis (ii) has the form:

$$ {Y}_{ijht}={\beta}_0+{\beta}_1 Pos{t}_{jh t}+{\beta}_2{x}_{ijht}+{\beta}_{3t}+{\beta}_{4h}+{\theta}_{jh}+{\varepsilon}_{ijht} $$

in which *Y*_*ijht*_ is the outcome variable for patient *i* seen by clinic *j* at hospital *h* at time *t* while *Post*_*jht*_ is a time-varying practice within hospital indicator of whether the intervention had been rolled-out in clinic *j* at hospital *h* by time *t* (1 = intervention transition has occurred by time period *t* and 0 = direct admission intervention has not occurred by time-period *t*) at the time when the observation is made. The other predictors are as defined for model (i). The key coefficient of interest is *β*_1_, which captures the structural shift in the outcome that occurs when a practice receives the direct admission intervention. Because *Post*_*jht*_ does not vary within a clinic and time-period, the information in the observations made within a clinic and time-period depends on the size of the effects of clinic and time-period and the statistical significance of inference about *β*_1_ is likely to be reduced by the clustering of observations in clinics. An additional component of clustering arises because the four groups are aligned with nine practice groups that in turn are embedded in three hospitals; clustering is of greater concern for this analysis as the key predictor only varies by cluster-time units, not among individuals within cluster-time units. We acknowledge that there may be separate levels of clustering in the data due to hospital and practice groups. However, the nine practice groups emanating from the 3 hospitals will be randomly distributed across the four groups of the stepped wedge design in a manner that balances the four groups with respect to the characteristics of these groups. Because the practice groups and hospitals are cross-classified with the four groups in our stepped wedge design, their effects on the statistical precision of the results will be much less than under a purely hierarchical arrangement.

For aim 2, to test our hypothesis that children with UTI, SSTI, and complex chronic conditions will experience the greatest benefits from direct admission relative to children admitted with other clinical diagnoses, we will evaluate group-level heterogeneity of treatment effects (HTE) by conducting subgroup analyses as specified a priori. Although there is no prior literature about the relative benefits of direct admission for these subgroups, this hypothesis is derived from our deliberative discussions with multidisciplinary stakeholders, based on their lived experiences. Our anticipated sample size for subgroups is derived from national statistics about pediatric inpatient stays, shown in Table 1. Although our assessment of HTE will follow methodological guidance that all subgroups be specified a priori, we will consider other subgroup analyses based on recommendations from Parent Partners and hospitals’ DALTs [25, 26]. Subgroup analyses will be performed by estimating an analogous model to that for the above-described patient-level analysis. To evaluate whether the effect of the direct admission intervention varies significantly across subgroups, we will augment the model specified in the aim 1 analysis with a predictor for the product of the subgroup-defining variable and direct admission; the coefficient of the resulting variable is referred to as an interaction effect and its value captures the extent to which the effect of direct admission differs between one level of the subgroup to the other. This approach is based on the recommendations by Kent et al., who recommend analysis and reporting of multivariate risk-based HTE to account for the fact that patients have multiple characteristics simultaneously that affect the likelihood of benefiting from an intervention [26, 27].

**Table 1:** Components of mixed methods process evaluation using RE-AIM Framework

Domain and definition	Approach
Reach: the % and characteristics of children eligible for the direct admission intervention who were admitted via this approach	Monthly reports to primary care practices reporting the number and % of eligible children admitted via direct admission; quarterly reports displaying clinical and sociodemographic characteristics of children admitted directly compared to those admitted via the ED
Efficacy: consideration of positive and negative outcomes of the intervention	Quarterly reports to primary care practices of primary and secondary study outcomes
Adoption: barriers to and facilitators of adopting this intervention	Qualitative interviews with key stakeholders will focus on (i) experience with the direct admission intervention and (ii) barriers to and facilitators of (a) referral for direct admission, (b) delivery of the intervention, including adherence to core components, (c) provision of timely and patient-centered care, and d) assurance of patient safety
Implementation: the extent to which the intervention is delivered as intended	Qualitative interviews with stakeholders, and quarterly meetings of the Direct Admission Leadership Teams to discuss barriers to and facilitators of adherence to intervention
Maintenance: the extent to which the intervention is sustained over time	Monthly reports to primary care practices regarding the number and % of eligible children admitted via direct admission, demonstrating changes over time

Lastly, for aim 3, in order to determine the impact of our direct admission intervention and to inform post-project implementation in other health systems, we will conduct a mixed-methods process evaluation applying the RE-AIM implementation framework to assess reach, effectiveness, adoption, implementation, and maintenance of our direct admission intervention [28]. These domains, definitions, and our proposed approach are summarized in Table 2. This approach combines analysis of our primary and secondary outcomes with analysis of process measures and qualitative interviews with stakeholders. Semi-structured interviews focused on the adoption, implementation, and maintenance domains will be conducted with parents of hospitalized children, referring PCPs, and inpatient healthcare team members including nurses, resident physicians, hospitalists, and other key stakeholders. We anticipate completing 12–16 in-depth interviews with stakeholders at each hospital system each year, for a total of 72–96 interviews over the 2-year implementation period. However, interviews will be continued until thematic saturation is reached [29]. Interviews will be recorded with permission, transcribed verbatim, and analyzed iteratively using a general inductive approach [30–32].

**Table 2:** Projected subgroup sample sizes

Condition/population (total sample ***n*** = 1696)	Anticipated, ***n*** (%)
Medical complexity	
No chronic disease	644 (38%)
Chronic, non-complex	441 (26%)
Complex chronic disease	611 (36%)
Diagnosis	
Pneumonia	551 (33%)
Skin/soft tissue infection	262 (16%)
Gastroenteritis/dehydration	317 (19%)
Urinary tract infection	182 (11%)
Viral infection not otherwise specified	293 (18%)
Influenza	51 (3%)

### Sample size and statistical power

**Fig. 3:**
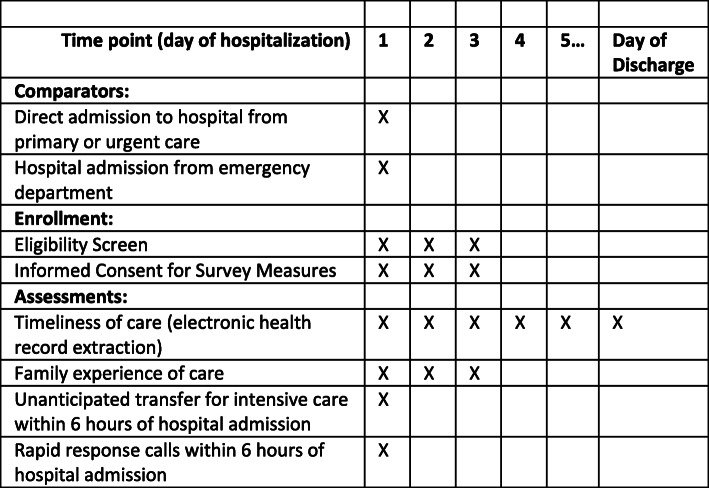
Schedule of comparators, enrollment, and assessments, all initiated on the day of arrival at the hospital

Based on the review of administrative data from participating sites, we anticipate that 190 children and adolescents will be directly admitted, with 1506 admitted through the ED over the 24-month implementation period (Figs. 2 and 3). We expect full data (100% of study participants) for our primary outcome, which will be derived from EHR data available for all hospitalized children by hospital protocol and procedures. This is also true of our secondary outcomes derived from EHR data, including ICU transfer and rapid response calls. However, for our parent-reported measure of family experience of care, we estimate a response rate of 75%, accounting for potential parental refusals, absence of parents from the bedside, and inability to complete the survey in English, Spanish, Arabic, Nepali, or Somali [33, 34]. Of those admitted through EDs, we estimate that 870 will be during post time-periods. Therefore, for the patient-level analysis described above, we anticipate 190 direct admissions and 870 concurrent control ED admissions.

Because the effects of all forms of clustering in the patient level observational analysis of aim 1 are minimal, we illustrate the statistical power for this analysis by using a two-group *t* test. To inform our power calculations, we collected pilot data from a pediatric hospital medicine program with a well-established direct admission program, applying similar eligibility criteria and definitions proposed for this study. In this pilot study, we found that mean time to clinical care for ED admissions was 85 min [SD 83 min], and mean time for direct admissions was 40 min [SD 28 min].

With the above means and standard deviations, a two-sided 0.05-level test that allows for unequal standard deviations performed in the context of the patient level observational analysis of aim 1 has a power greater than 0.99. There is also substantial power for subgroup analyses. For example, the power of a subgroup analysis that involves 10% of the sample (applied evenly across clinics and time-intervals) has a power of 0.98. It is only when the subgroup is as small as 5% that power falls below 0.80. This power calculation was performed using Satterwaite’s approximation (i.e., evaluating a pooled variance to approximate the sampling distribution of the actual test-statistic by a t-distribution) under nQuery [35, 36].

For the system-level stepped wedge analysis, we compute power for the effect of the predictor labeled post, the effect of a clinic receiving the intervention and entering the post-period, for the analysis of the entire sample. The method of computing power for a design with unequal standard deviations in the prior and post time-periods is to first determine the design-effect for the stepped wedge design and then compute the power for a two-population comparison using the effective-sample-sizes based on the design-effect. The design-effect is estimated using the expression in Woertman et al. (2013), also described in Hemming (2016) [37, 38]. Because hospital and practice group are cross-classified across the four stepped wedge design groups, and because little is known about likely levels of interclass correlation coefficients (ICC) for each of hospital, practice group, and clinic, we perform illustrative power calculations assuming that the net impact of clustering is equivalent to hospital-level clustering alone with an ICC of hospital of 0.05. Based on results for past cluster-randomized studies at the clinic level, an intraclass correlation of 0.05 appears to be a conservative estimate of clustering by clinic [39, 40]. It is not unreasonable for 0.05 to still be conservative once the upper-level sources of cross-classified clustering are absorbed. The other inputs are 70 practice sites (clusters), 4 waves of intervention onset, no-baseline period, 1696 patients in total and equal numbers of patients per clinic per time-interval. Under this scenario, the design-effect is 2.385. Therefore, the effective sample size (ESS) is 711.

The second part of the calculation is to determine the power of a two-group comparison of a continuous outcome in the absence of clustering when the total sample size is 711. Because there are 10 group times in the post-period and 6 in the pre-period each with 106 observations, the split in the ESS between the post and the pre-period is 444 and 267. The mean and standard deviation of the outcome in the pre-period is given by the above values for ED admissions; the mean is 85 min and the standard deviation is 83. To determine the mean and standard deviation of the outcome expected in the post-period, we take weighted averages of the direct admission and ED admission means and standard deviations using the total theorem of probability to compute marginal effects. The weight of direct admission to the total patients is 19/106 = 0.094. The weighted average marginal mean and weighted average marginal standard deviation of the observations in the post-period are 76.93 and 84.99, respectively. Because the sample-sizes are reasonably large, an asymptotic normal approximation is well justified (i.e., even if the true distribution of the outcome is bimodal it will be averaged out and converging towards normality). Given the ESS’s and these means and standard deviations, a two-sided 0.05-level test has a power of 0.23. In order to have 0.80 power, we would need a much larger effect size. Specifically, the mean for the post-period would need to be approximately 66.5. Due to the small fraction of patients who are admitted directly, this will only be plausible if there is a spillover effect of the intervention such that the outcome is reduced for both patients admitted directly and via the ED.

### Data management

With the exception of our parent-reported outcome, all required data for this project will be extracted from the hospitals’ EHRs; all proposed variables exist in EHRs, thereby minimizing data collection burden and missingness as a result of non-response. During the pre-implementation period, all sites will conduct pilot data extraction from EHRs to assess data source adequacy; any deficiencies will be addressed prior to the rollout. A subset of records will be entered in duplicate at each site and checked for data quality. Following data extraction at hospital sites, de-identified data will be sent via HIPAA-secure methods to the research hub, where all data analyses will be conducted and subsequently shared with sites. Adverse events and other unintended effects will be communicated to the institutional review board and the funder as appropriate.

### Dissemination

All protocol amendments will be communicating with the institutional review board, the funder, and implementation sites. Public access to the full protocol, participant-level dataset, and statistical code will adhere to guidelines from the funder. We anticipate several peer-reviewed manuscripts and presentations resulting from this work; authorship decisions will adhere to guidelines by the International Committee of Medical Journal Editors (ICMJE). Additionally, we will disseminate our findings to our health system partners and via national organizations, social media, press releases, and media alerts [33].

## Discussion

Our study represents the first RCT to compare the effectiveness of direct and ED admission for children. We will implement a multi-stakeholder informed admission process and evaluate outcomes across diverse settings to advance our understanding of factors that support and hinder an effective direct admission process. Our scientific approach, pairing a robust design with a multi-level assessment of barriers to and facilitators of implementation, will generate valuable data to inform implementation of direct admission programs at other healthcare systems nationally.

A better understanding of the comparative effectiveness of direct versus ED admission is important to parents of hospitalized children and to their healthcare teams. Parents and physicians recognize potential benefits and challenges of direct and ED admission and articulate a desire for standardized admission processes [8, 16]. Consistent with the Institute of Medicine’s domains of healthcare quality, parents describe the importance of timely, effective, safe, and patient-centered clinical care, but express variation in their experiences with direct and ED admissions in achieving these aims [8]. In addition, the growth of pediatric hospital medicine in the US, the pediatric specialty dedicated to the inpatient care of children, creates both opportunities for around-the-clock pediatric-specific care but also creates a discontinuity of care between outpatient and inpatient providers [41–43]. Implementation of effective direct admission programs has the potential to improve patient hand-offs, communication between healthcare providers, efficiency and timeliness of care, and patient and family experience.

## Supplementary Information

**Additional file 1.** SPIRIT 2013 Checklist: Recommended items to address in a clinical trial protocol and related documents.

**Additional file 2.** CONSORT 2010 checklist of information to include when reporting a cluster randomised trial.

## Data Availability

Not applicable as no datasets were generated or analyzed for the production of this study protocol.

## Abbreviations

DALT: Direct Admissions Leadership Team
ED: Emergency department
EHR: Electronic health record
HTE: Heterogeneity of treatment effects
ICU: Intensive care unit
PCP: Primary care practice
PHM: Pediatric hospital medicine
RCT: Randomized controlled trial
SSTI: Skin and soft tissue infections
UTI: Urinary tract infections
